# A Comparison of the Lateral Approach (Paramedian) Versus the Modified Lateral Approach (Modified Paramedian) in Spinal Anesthesia: Evaluating Ease of Procedure and Patient Satisfaction in Urological Surgeries; A Triple-Blind Randomized Clinical Trial

**DOI:** 10.5812/aapm-161542

**Published:** 2025-05-26

**Authors:** Mehrdad Mesbah Kiaei, Siavash Sangi, Maryam Aligholizadeh, Mahmoud Reza Mohaghegh Dolatabadi, Ali Moshki, Mohsen Abbasi

**Affiliations:** 1 Department of Anesthesiology and Pain Medicine, School of Medicine, Hasheminejad Kidney Center, Iran University of Medical Sciences, Tehran, Iran; 2 Department of Anesthesiology and Operating Room, School of Nursing and Midwifery, Shahid Beheshti University of Medical Sciences, Tehran, Iran; 3 Department of Anesthesia, School of Medicine, And Hospital Management Research Center, Health Management Research Institute, Iran University of Medical Sciences, Tehran, Iran; 4 Department of Anaesthesia and Critical Care, Hasheminejad Hospital, School of Medicine, Iran University of Medical Sciences, Tehran, Iran

**Keywords:** Spinal Anesthesia, Paramedian Approach, Modified Paramedian Approach, Procedure Feasibility, Patient Satisfaction, Urological Surgeries

## Abstract

**Background:**

Spinal anesthesia (SA) is preferred over general anesthesia for lower extremity surgeries, but the optimal method of needle placement is debated. Although the paramedian approach reduces the risks of dural puncture, it presents technical difficulties. The modified paramedian technique may increase safety and patient satisfaction by facilitating subarachnoid access and overcoming anatomical challenges, particularly in obese or elderly patients.

**Objectives:**

This study aimed to compare the paramedian and modified paramedian techniques from the perspective of anesthesiologists and their impact on postoperative patient satisfaction.

**Methods:**

This triple-blind randomized clinical trial investigated the effects of two SA techniques — paramedian and modified paramedian — on patient satisfaction and procedural ease. A total of 112 patients meeting inclusion and exclusion criteria were enrolled. Data were collected using the Iowa Satisfaction with Anesthesia Care Questionnaire. Demographic information was recorded in coded form, and data analysis was performed using SPSS version 19. Statistical methods included the independent *t*-test for comparing continuous means between groups, the chi-square test for categorical variables, and logistic regression analysis to assess the impact of individual characteristics (age, gender, weight) on the ease of performing spinal anesthesia.

**Results:**

The results indicated that the modified paramedian group demonstrated superior performance in terms of success on the first attempt (P = 0.006), reduced need for repositioning (P = 0.038), and fewer repeated attempts (P = 0.017). Additionally, patient satisfaction scores were significantly higher in the modified paramedian group (P = 0.001). Multivariate regression confirmed age and Body Mass Index (BMI) as independent predictors of procedural difficulty (P < 0.05).

**Conclusions:**

The modified paramedian technique significantly enhanced the ease of SA administration and patient satisfaction compared to the traditional approach. These findings indicate its potential to improve the anesthesia process, reduce side effects, and elevate patient experience. This study supports broader adoption of the technique in surgical and healthcare settings, advancing anesthesia care quality.

## 1. Background

Spinal anesthesia (SA) is a key technique used in lower limb and urological surgeries. It offers several advantages, including rapid onset, predictable duration, and ease of administration (1). Compared to general anesthesia, SA has been linked to better patient outcomes, such as higher satisfaction rates, reduced opioid use, and lower postoperative pain scores. These benefits make it a preferred choice in many clinical settings (2). Furthermore, SA is associated with a reduced incidence of thromboembolic events and a diminished need for perioperative blood transfusions, further emphasizing its clinical benefits (3). While the midline approach is commonly used, the paramedian approach is often considered in cases where anatomical challenges, such as obesity or degenerative spinal changes, make the midline approach difficult (4). The paramedian technique, particularly in elderly patients, has been shown to reduce procedure time and improve success rates, though it requires greater technical skill and spatial awareness (3, 5). Recognizing the limitations and technical demands of the conventional paramedian approach, we have developed a modified paramedian technique aimed at addressing these challenges. This modified approach integrates subtle adjustments in needle angulation and entry points to optimize the procedural trajectory and minimize technical difficulty. By refining the approach, this modification is expected to increase the ease of prescribing and overall patient experience. The present study seeks to conduct a comparative evaluation of the paramedian and modified paramedian approaches, with a focus on procedural efficiency and patient satisfaction, and the midline approach is not under investigation.

## 2. Objectives

This investigation aims to contribute to the ongoing discourse on the optimal technique for spinal anesthesia, providing evidence-based insights to guide anesthetic practice in urological surgeries.

## 3. Methods

### 3.1. Design and Settings

This triple-blind randomized clinical trial was registered with the Iranian Registry of Clinical Trials (IRCT20161220031487N10) and received ethical approval from the Ethics Committee of Iran University of Medical Sciences (IR.IUMS.FMD.REC.1403.194). The study was conducted in hospitals affiliated with Iran University of Medical Sciences, with the primary outcomes being the ease of the procedure and patient satisfaction. It spanned five months from June 2024 to March 2025.

### 3.2. Eligibility Criteria for Participants

Patients aged 18 - 70 years, classified as ASA I, II, or III, were eligible for inclusion. Exclusion criteria included contraindications to SA (e.g., infection at the injection site), recent use of anticoagulants, obesity [Body Mass Index (BMI) > 38 kg/m^2^], uncontrolled diabetes or hypertension, severe cardiac valve disease, neurological or psychiatric disorders, substance or alcohol abuse, recent corticosteroid use, excessive bleeding, surgeries lasting over three hours, or anesthesiologists with less than five years of experience. After obtaining ethical approval, detailed information about the study was provided, and written informed consent was secured from all participants.

### 3.3. Sample Size

The sample size calculation was conducted using G Power 3.1 software with a confidence level of 95% and a power of 80%. The assumed difference in mean values between groups was μ1 - μ^2^ = 1.7, with a standard deviation σ = 0.9 and a drop rate of 10%. A total of 112 patients were recruited and equally randomized into two groups: Fifty-six in the paramedian approach group (Group P) and 56 in the modified paramedian approach group (Group MP). It should be noted that the estimated standard deviation was based on previous studies (6).

### 3.4. Randomization

Regarding the sampling method, eligible participants were recruited from among the patients and were randomly assigned to two groups using block randomization. Randomization was performed using four-block sequences (e.g., ABAB, BAAB, etc.) generated in Excel. Sequence codes were sealed in envelopes and managed by an independent epidemiologist to ensure allocation concealment.

### 3.5. Blinding

A triple-blind design was employed to reduce bias and ensure the validity of the results. Patients were randomly assigned to one of two groups without knowledge of the specific SA technique used (paramedian or modified paramedian). The researcher collecting data and the statistician analyzing the results were also blinded to group assignments. Additionally, nurses in the recovery and operating rooms were blinded to the group assignments. Only one clinical observer was considered, and the observer was trained to standardize assessments. This study employed a triple-blind approach, ensuring that patients, data collectors/evaluators, and the statistical analyst were unaware of the prescribed medication.

### 3.6. Data Collection Tools

Patient satisfaction was assessed using the validated Iowa Satisfaction with Anesthesia Scale (ISAS), which consists of 11 sections on a 6-point Likert scale: Three questions related to pain, six questions about experiences during anesthesia, and two questions directly assessing satisfaction with anesthesia (7). Therefore, the highest score for patient satisfaction with Iowa anesthesia care is 66, and the lowest score is 11. Overall, the purpose of this questionnaire was to collect information about the patient's experience during spinal anesthesia, postoperative symptoms, and their satisfaction with the procedure. The ISAS has demonstrated strong reliability (Cronbach’s alpha = 0.716) and validity (CVR = 80%, CVI = 0.80) (8).

The ease of the procedure was evaluated based on the following criteria:

(1) Easy: Successful dural puncture and cerebrospinal fluid (CSF) visualization on the first attempt.

(2) Moderate: Successful dural puncture after one or two attempts or repositioning within the same or adjacent intervertebral space.

(3) Difficult: Three or more attempts or the need for alternative anesthesia techniques.

The effectiveness of SA was determined by factors such as failed attempts beyond three, the need for additional analgesics, or general anesthesia due to patient intolerance (9). This classification standardizes SA difficulty assessment based on CSF visibility, attempt count, and repositioning needs. Patient data, including demographics, surgery details, and ASA classification, were recorded using a coded checklist. Vital signs were monitored at defined intervals, and hemodynamic complications, such as postoperative headaches, were documented. To reduce bias, only specialists with over five years of experience performed the procedures.

### 3.7. Surgical Procedure

Upon entering the operating room, the temperature was maintained at an optimal range of 22 - 24°C in both the operating and recovery areas. An 18 - 20 gauge intravenous line was established for all patients. All intravenous fluids administered to patients were maintained at room temperature. No preoperative sedation was administered. Before initiating spinal anesthesia, all patients received a preload of 5 - 6 mL/kg of normal saline, which was continued intraoperatively at a rate of 7 - 8 mL/kg/hour. Throughout the procedure, patients were monitored using standard cardiopulmonary monitoring tools, including a 5-lead electrocardiogram (ECG), non-invasive blood pressure (NIBP) monitoring, and pulse oximetry. Baseline vital signs, including body temperature, heart rate (HR), blood pressure, respiratory rate (RR), and oxygen saturation (SpO_2_), were recorded.

#### 3.7.1. Group P (Paramedian Approach)

The SA was performed at the L3 - L4 or L4 - L5 interspace using a 27-gauge Quincke needle with the patient in the sitting position. The needle was inserted 1 cm lateral and 1 cm caudal to the spinous process, directed at a 10 - 15° angle cephalad and medially.

#### 3.7.2. Group MP (Modified Paramedian Approach)

**Figure 1.**
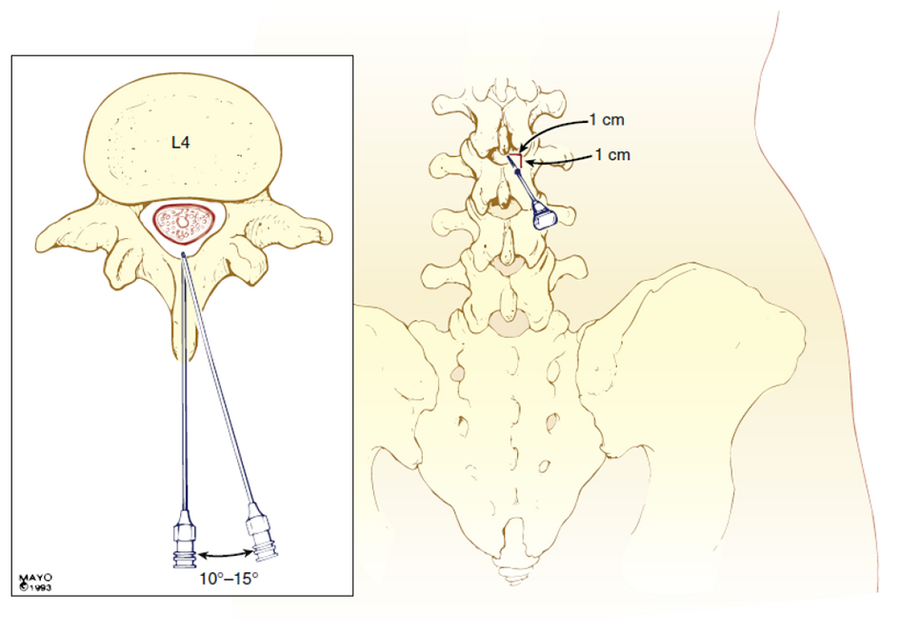
Paramedian approach for spinal anesthesia (SA) (10)

**Figure 2.**
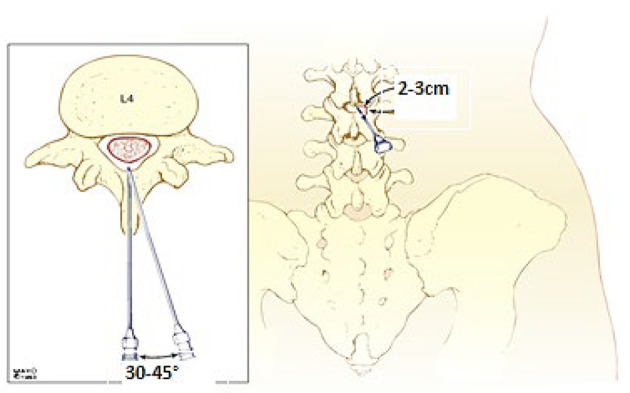
Modified paramedian approach for spinal anesthesia (SA)

Patients were positioned sitting, and using a 27-gauge Quincke needle, the needle was inserted 2 - 3 cm lateral and 2 - 3 cm caudal to the spinous process, directed at a 30 - 45° angle cephalad and medially (Figures 1 , 2, and Table 1). 

**Table 1.** Comparison of Spinal Anesthesia Techniques

Parameters	Group P (Paramedian)	Group MP (Modified Paramedian)
**Position**	Sitting	Sitting
**Needle gauge/type**	27G Quincke	27G Quincke
**Insertion point**	1 cm lateral & caudal to spinous process	2 - 3 cm lateral & caudal to spinous process
**Needle angle**	10 - 15° (cephalad & medial)	30 - 45° (cephalad & medial)
**Drug administration**	2 - 3 mL 0.5% hyperbaric bupivacaine	2 - 3 mL 0.5% hyperbaric bupivacaine
**Oxygen delivery**	6 L/min facemask	6 L/min facemask
**Assessments**	Block level (5 min), ease of procedure, satisfaction	Block level (5 min), ease of procedure, satisfaction

In both groups, 2 - 3 mL of 0.5% hyperbaric bupivacaine was administered intrathecal. Oxygen was delivered at a rate of 6 L/min via a facemask throughout the procedure. Sensory and motor block levels were assessed 5 minutes after SA administration. The ease of the procedure was evaluated by an independent observer blinded to the study groups, using predefined criteria. Patient satisfaction was assessed using a validated questionnaire at four time points: Before surgery, at the end of surgery, upon arrival in the recovery room, and at discharge from the recovery room. The assessments were conducted by an observer blinded to the study groups.

### 3.8. Statistical Analysis

Descriptive statistics, including mean, standard deviation, frequencies, and percentages, were used to summarize the data. The chi-square test was applied for qualitative outcomes, and logistic regression analysis was used to assess the impact of individual characteristics (age, gender, weight) on the ease of performing spinal anesthesia, with significance set at P < 0.05. For continuous variables, independent samples *t*-tests were used, while non-parametric data were analyzed using the Mann-Whitney U test. All analyses were performed using SPSS version 19.

## 4. Results

**Figure 3.**
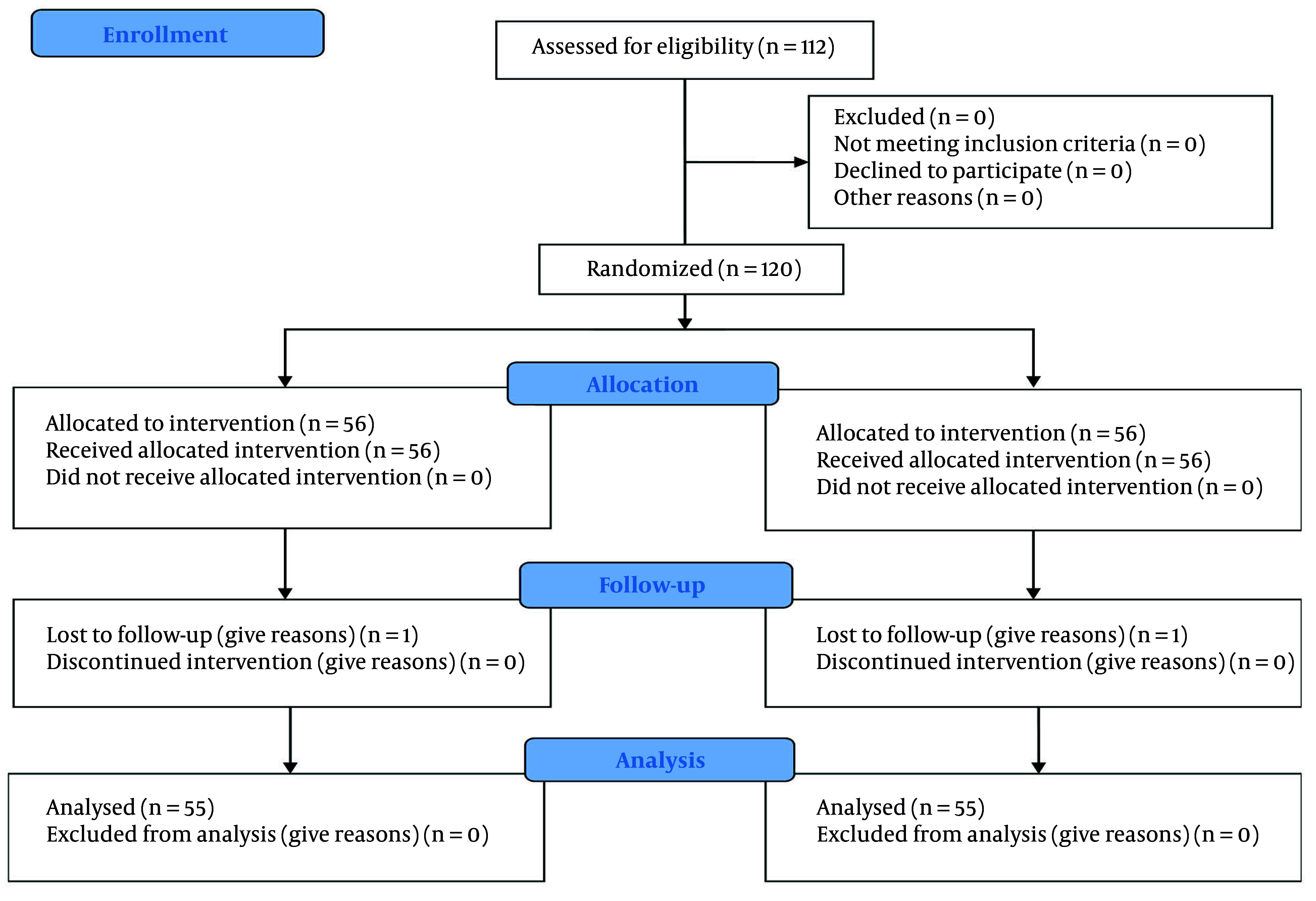
CONSORT flow diagram

A total of 112 patients eligible for urological surgeries were enrolled in the study based on the inclusion and exclusion criteria. During the course of the study, one patient in the intervention group was excluded due to the prolongation of the surgery, which resulted in a conversion to general anesthesia, and one patient in the control group was excluded due to the cancellation of the surgery (Figure 3). There were no significant differences between the two groups regarding demographic characteristics such as age, gender, BMI, and other individual traits (P > 0.05) (Table 2). 

**Table 2.** Comparison of Demographic Characteristics Between the Two Groups ^a^

Variables	Paramedian (N = 56)	Modified Paramedian (N = 56)	Statistical Test	P-Value^b^
**Age**	60.1 ± 5.2	59.3 ± 4.1	Independent *t*-test	0.78
**Gender (male - female)**	22 - 34	20 - 36	Chi-square test	0.68
**Body Mass Index**	28.1 ± 6.0	27.3 ± 2.2	Independent *t*-test	0.55
**ASA class I/II**	14/42	16/40	Chi-square test	0.73
**History of comorbidities**	20 (35.7)	18 (32.1)	Chi-square test	0.65

^a^ Values are expressed as Mean ± SD, No. (%) or No.

^b^ P < 0.05 was considered statistically significant.

The feasibility of performing spinal anesthesia, as assessed by anesthesia specialists, demonstrated significant differences between the paramedian and modified paramedian approaches. In the modified paramedian group, success on the first attempt was significantly higher (85.7% vs. 60.7% in the paramedian group, P = 0.006). Additionally, the need for patient repositioning was lower in the modified paramedian group (7.1% vs. 21.4%, P = 0.038), and the requirement for repeated attempts (≥ 2 attempts) was also reduced (3.6% vs. 17.9%, P = 0.017). The visualization of CSF on the first attempt was higher in the modified paramedian group (89.3% vs. 64.3%, P = 0.003). Furthermore, the mean ease-of-performance score (out of 10) was significantly higher in the modified paramedian group (8.6 ± 1.2 vs. 6.5 ± 1.4, P = 0.002) (Table 3). 

**Table 3.** Comparison of the Ease of Performing Spinal Anesthesia as Assessed by Anesthesiologists in the Two Study Groups ^a^

Ease of Spinal Anesthesia Performance Indicators	Paramedian (N = 56)	Modified Paramedian (N = 56)	P-Value ^b^
**Success on the first attempt**	34 (60.7)	48 (85.7)	0.006
**Need for repositioning**	12 (21.4)	4 (7.1)	0.038
**Need for repeated attempts (≥ 2 attempts)**	10 (17.9)	2 (3.6)	0.017
**Visualization of cerebrospinal fluid on the first attempt **	36 (64.3)	50 (89.3)	0.003
**Mean ease-of-performance score (out of 10)**	6.5 ± 1.4	8.6 ± 1.2	0.002

^a^ Values are expressed as Mean ± SD or No. (%).

^b^ P < 0.05 was considered statistically significant.

Comparison of patient satisfaction scores after SA between the paramedian and modified paramedian groups revealed significantly higher overall satisfaction scores in the modified paramedian group (58.7 ± 4.3 vs. 48.8 ± 4.5, P = 0.001). Regarding individual indicators, the absence of nausea and vomiting was improved in the modified paramedian group (2.8 ± 0.5 vs. 2.2 ± 0.7, P = 0.012). Additionally, the absence of pain during surgery was better in this group (2.9 ± 0.4 vs. 2.4 ± 0.6, P = 0.001). Patients in the modified paramedian group also reported higher levels of calmness (2.7 ± 0.6 vs. 2.1 ± 0.8, P = 0.023) and security (2.8 ± 0.5 vs. 2.3 ± 0.7, P = 0.017). Furthermore, patients in this group experienced fewer extreme sensations of cold or heat (2.9 ± 0.4 vs. 2.5 ± 0.6, P = 0.034). The likelihood of choosing the same method again was also significantly higher in the modified paramedian group (2.9 ± 0.3 vs. 2.3 ± 0.7, P = 0.001) (Table 4). 

**Table 4.** Comparison of Patient Satisfaction Scores After Spinal Anesthesia in the Two Study Groups ^a^

Satisfaction Indicators	Paramedian (N = 56)	Modified Paramedian (N = 56)	P-Value
**Overall satisfaction score (out of 66)**	48.5 ± 4.8	58.3 ± 4.5	0.001 ^b^
**Absence of nausea and vomiting**	2.2 ± 0.7	2.8 ± 0.5	0.012
**Absence of pain during surgery**	2.4 ± 0.6	2.9 ± 0.4	0.001 ^b^
**Feeling of calmness**	2.1 ± 0.8	2.7 ± 0.6	0.023
**Feeling of security**	2.3 ± 0.7	2.8 ± 0.5	0.017
**No extreme sensation of cold or heat**	2.5 ± 0.6	2.9 ± 0.4	0.034
**Likelihood of reusing the method**	2.3 ± 0.7	2.9 ± 0.3	0.001 ^b^

^a^ Values are expressed as Mean ± SD.

^b^ P < 0.05 was considered statistically significant.

To control for potential confounding factors, a multivariate logistic regression analysis was performed, with the ease of SA (categorized as Easy vs. Moderate/Difficult) as the dependent variable, and age, BMI, and gender as independent variables. The multivariate analysis revealed that higher age (adjusted OR = 1.45, 95% CI: 1.12 - 1.87, P = 0.003) and higher BMI (adjusted OR = 1.39, 95% CI: 1.07 - 1.81, P = 0.009) were independently associated with greater difficulty in performing spinal anesthesia. Gender was not a significant predictor (adjusted OR = 1.13, 95% CI: 0.79 - 1.62, P = 0.48). These findings confirm that even after adjusting for other factors, age and BMI remain strong predictors of procedural difficulty (Table 5). 

**Table 5.** Association Between Individual Characteristics and the Ease of Spinal Anesthesia Administration

Variables	Adjusted Odds Ratio (OR)	95% Confidence Interval (CI)	P-Value	Interpretation
**Age (y)**	1.45	1.12 - 1.87	0.003 ^a^	Higher age → greater difficulty
**BMI (kg/m** ^**2**^ **)**	1.39	1.07 - 1.81	0.009 ^a^	Higher BMI → greater difficulty
**Gender (male = 1, female = 2)**	1.13	0.79 - 1.62	0.48	No significant effect

Abbreviation: BMI, Body Mass Index.

^a^ P < 0.05 was considered statistically significant.

## 5. Discussion

The SA remains the preferred choice for lower extremity and urological surgeries due to its superior hemodynamic stability, reduced postoperative pain, and lower incidence of systemic complications compared to general anesthesia (11). However, the choice of technique for needle placement continues to be a subject of debate, particularly in patients with anatomical variations such as obesity, spinal degeneration, or advanced age (12). This triple-blind randomized clinical trial provides compelling evidence that the modified paramedian approach to SA outperforms the conventional paramedian technique in both procedural ease and patient satisfaction during urological surgeries. Our results suggest that the modifications to needle insertion angle and positioning may facilitate subarachnoid space access and reduce the technical challenges encountered in the conventional paramedian approach.

The modified paramedian approach significantly improves technical success rates, reducing the need for multiple attempts and repositioning. This aligns with previous findings by Cormican (13), who reported that alternative SA techniques can mitigate the common technical difficulties encountered with the traditional midline and paramedian approaches. The modified technique likely facilitates a more direct and predictable trajectory to the subarachnoid space, bypassing calcified interspinous ligaments and reducing procedural failures.

A multivariate logistic regression model was developed to adjust for potential confounders. This analysis confirmed that age and BMI were independently associated with increased difficulty of spinal anesthesia, even after adjusting for other factors. These results emphasize the importance of considering patient characteristics when selecting and performing SA techniques (14, 15). The anatomical alterations associated with obesity, including increased lumbar lordosis and soft tissue obstruction, often necessitate advanced techniques to achieve reliable subarachnoid access. The modified paramedian approach, by circumventing these obstacles, appears to offer a more effective solution in such patient populations. The results align with prior research indicating that paramedian approaches are particularly advantageous in patients with difficult spinal anatomy, such as those with obesity or degenerative spinal changes (16). However, while conventional paramedian techniques have been shown to increase procedural success rates compared to the midline approach, they often require greater technical skill (2). This study contributes novel insights by demonstrating that the modified paramedian technique further refines the procedural approach, leading to even greater success rates while maintaining ease of execution. A previous study by Chen et al. highlighted that learning curves associated with conventional paramedian techniques can be steep for novice anesthesiologists (17). The modified paramedian approach in this study may help mitigate some of these challenges by providing a more predictable trajectory for needle insertion.

Additionally, Arslan & Şahin found that the paramedian technique should be the first choice for geriatric patients due to its reduced risk of dural puncture failure (5). The current findings extend this evidence by demonstrating that further modification of the paramedian approach enhances procedural efficiency across a broader patient population. On the other hand, our study findings were largely consistent with expectations, as previous literature suggests that paramedian approaches facilitate easier needle placement in patients with challenging anatomical variations (6). However, one surprising finding was the degree to which patient satisfaction improved in the modified paramedian group. The significantly higher satisfaction scores, including a greater likelihood of patients choosing the same method again (P = 0.001), suggest that the procedural improvements have meaningful patient-centered benefits beyond just technical success rates.

Patient satisfaction represents a critical metric in perioperative care, influencing overall surgical outcomes and hospital quality assessments. The significantly higher Iowa Satisfaction with Anesthesia Care scores in the modified paramedian group (P = 0.001) strongly indicate that improved procedural efficiency translates into better patient experiences. Prior studies have established that reducing the number of puncture attempts and procedural duration enhances patient comfort and minimizes anxiety (18, 19). Furthermore, since repeated dural punctures are associated with complications such as post-dural puncture headache (PDPH) and back pain, the observed reduction in needle repositioning and multiple attempts suggests that the modified technique may also contribute to lower postoperative complication rates, although this warrants further longitudinal investigation.

Moreover, while prior studies indicated that factors such as age and BMI influence the difficulty of SA (9), the current study reinforced these associations, demonstrating a statistically significant correlation between these factors and procedural difficulty. This reinforces the need for anesthesia providers to tailor their approach based on patient characteristics. The findings support the broader adoption of the modified paramedian technique in clinical practice. The improved ease of administration and enhanced patient experience suggest that this approach could become the preferred method for spinal anesthesia, particularly in urological surgeries and among patients with anatomical challenges. Furthermore, the reduction in repeated attempts and need for repositioning may decrease the risk of complications such as post-dural puncture headache, thereby improving postoperative outcomes and reducing hospital resource utilization (3).

This study has several limitations. The exclusion of patients with BMI > 38 kg/m^2^ limits generalizability to morbidly obese individuals, who may benefit from the modified paramedian approach. Also, its focus on urological surgeries restricts applicability to other procedures, such as cesarean or lower limb surgeries. The sample size (n = 112) and single-center design may overlook rare complications and reduce external validity, while the one-week follow-up may miss delayed events like back pain or neurological issues. Broader, longer-term studies are needed to confirm efficacy and safety. Future studies should evaluate the long-term effects of this approach on complication rates, particularly in high-risk populations such as geriatric or obese patients. Additionally, research comparing the modified paramedian approach with ultrasound-assisted SA techniques (2) could further clarify the optimal technique for achieving both technical success and patient satisfaction.

Given the robust evidence supporting its advantages, the modified paramedian approach should be considered for broader implementation in SA protocols, particularly for high-risk patient populations, including those with advanced age, obesity, or difficult spinal anatomy. Future research should explore the long-term safety profile, complication rates, and cost-effectiveness of this approach in larger multicenter trials to validate its generalizability. Overall, this study contributes substantially to the ongoing refinement of SA techniques, reinforcing the clinical superiority of the modified paramedian approach. By enhancing procedural success and optimizing patient-centered outcomes, this technique holds the potential to reshape modern anesthetic practice, ensuring safer and more efficient care for patients undergoing spinal anesthesia.

### 5.1. Conclusions

The present study, which compared the modified paramedian approach with the classical paramedian technique in SA for urological surgeries, demonstrated that the modified method significantly facilitates the anesthesia process and enhances patient satisfaction. This approach was associated with a reduction in side effects such as pain and discomfort, improved ease of execution for anesthesiologists, and an overall enhancement of the patient experience. Additionally, its use led to a decrease in physical injuries, postoperative disabilities, and treatment costs. The findings suggest that the modified paramedian technique can serve as an effective strategy for SA in other surgical procedures and healthcare settings, paving the way for future research aimed at refining anesthesia techniques. This study underscores the importance of adopting a holistic perspective in medical processes to improve patient health outcomes while reducing costs. By emphasizing both clinical efficacy and patient-centered care, the modified paramedian approach represents a significant advancement in the field of regional anesthesia.

### 5.2. Recommendations

This study’s strengths include its rigorous triple-blind design, robust statistical analysis, and focus on both procedural success and patient experience. Notably, it also identifies confounding variables, effectively minimizing bias. However, further research is needed to evaluate the modified paramedian technique in patients with anatomical variations (e.g., scoliosis, spinal degeneration) and across other surgical fields such as orthopedics and obstetrics. Expanding these investigations will enhance the generalizability and clinical applicability of the findings.

## Data Availability

The raw data and materials are available from the corresponding author upon reasonable request.

## Clinical Trial Registration Code:

IRCT20161220031487N10.
